# Supplementary material for: A Combined Angelica gigas and Artemisia dracunculus Extract Prevents Dexamethasone-Induced Muscle Atrophy in Mice through the Akt/mTOR/FoxO3a Signaling Pathway
Source: Cells. 2022 Oct 15;11(20):3245. doi: 10.3390/cells11203245 (PMC9600779; doi:10.3390/cells11203245)

Table S1. Total polyphenol and flavonoid contents of CHDT

Total phenolic content of CHDT was estimated with reference to the Folin Ciocalteu method. CHDT was mixed with 10% sodium carbonate, and 2% Folin Ciocalteu reagent to make a mixture. The mixture was incubated at room temperature for 1 h and the absorbance was measured at 750 nm by spectrophotometer (Molecular Devices Sunnyvale, CA, USA). Gallic acid was used as the standard and the total polyphenol content was expressed as milligram gallic acid equivalent (GAE) per gram of extract (mg GAE/g extract), the total phenolic content of CHDT was 18.94 ± 0.07 mg GAE/g. Total flavonoid content of CHDT was estimated by slightly modifying the method described in Udayaprakash et al., 2015. 0.1 mL of 10% aluminum chloride, 1.5 mL of 95% ethanol, 0.1mL of 1 M potassium acetate, 2.8 mL of distilled water was added to 500 µl of the extract to make a mixture. After incubating the mixture for 30 min at room temperature, the absorbance was measured at 415 nm by spectrophotometer. Rutin was used as the standard and the total flavonoid content was expressed as milligram quercetin equivalent (RE) per gram of extract (mg RE/g extract), the total flavonoid content of CHDT was 12.27 ± 0.35 mg RE/g.

Table S1. Total polyphenol and flavonoid contents of CHDT

	Total phenolic content (mg GAE/g)	Total flavonoid content (mg RE/g)
CHDT	18.94 ± 0.07	12.27 ± 0.35

Table S2. Other phenolic compounds analysis of CHDT

High performance liquid chromatography (HPLC) was equipped with Waters E2695 Separations Module HPLC and Waters 996 Photodiode Array Detector. CHDT was monitored by a PDA detector (Waters). CHDT was separated on a Sunfire TM C18 (250×4.6 mm, 5 μm, Waters). CHDT was dissolved with methanol, and the injection volume was 10 μL. Standards 9 phenolic compounds; catechin, chlorogenic acid, p-coumaric acid, ferulic acid, hesperidin, naringin, quercetin hydrate, rutin hydrate, vanillic acid for HPLC analysis were obtained from Sigma–Aldrich Co. (St. Louis, MO, USA). To detect 9 phenolic compounds, the mobile phase was composed of acetonitrile (ACN) (solvent A) and DW with 0.5% (v/v) Phosphoric Acid (H_3PO_4) (solvent B). The gradient program was 0-23 min, 8% B; 23-26 min, 8-15% B; 26-36 min, 15-30% B; 36-40 min, 30-45% B, 40-43 min, 45% B; 43-45 min, 45-8% B; 45-53 min, 8% B at a flow rate of 1.0 mL/min, and UV detection was performed at 280 nm. As a results, 3 of 9 phenolic compounds, chlorogenic acid, rutin, and ferulic acid, were detected in CHDT, and the contents of each are described in Table S1.

Table S2. Other phenolic compounds detected in the CHDT

Compounds	Contents (mg/dry weight g)
Chlorogenic acid	22.93 ± 0.16
Rutin	15.80 ± 0.14
Ferulic acid	0.29 ± 0.001

Figure S1. The effect of CHDT on hepatotoxicity. The serum levels of (**A**) aspartate transaminase (AST) and (**B**) alanine transaminase (ALT). There were no statistically significant difference between groups.

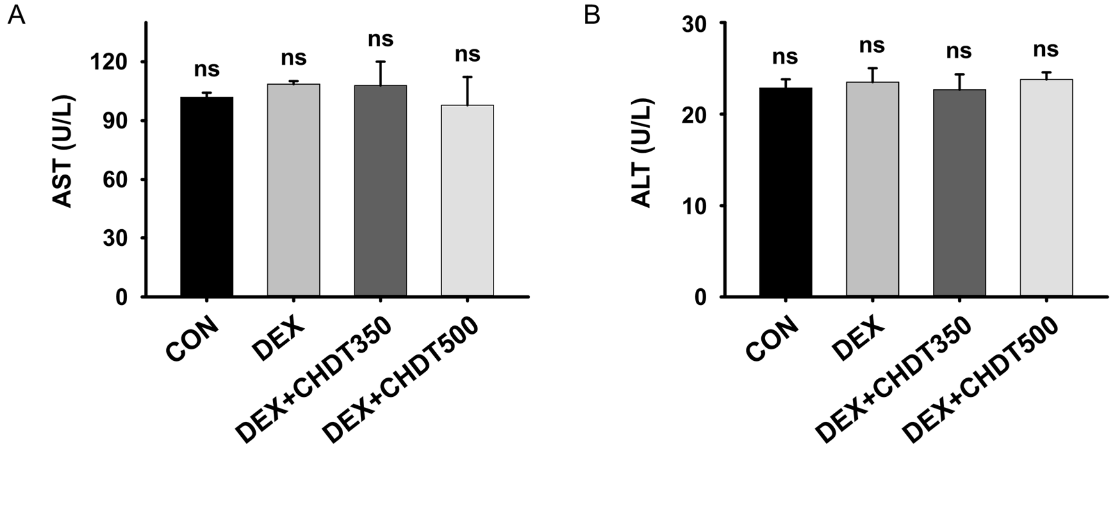

Supplement: Supplementary file 1 [file cells-11-03245-s001.zip › cells-1946817-supplementary.pdf]
